# PFF-CB: Multiscale Occlusion Pedestrian Detection Method Based on PFF and CBAM

**DOI:** 10.1155/2022/3798060

**Published:** 2022-04-21

**Authors:** Guiyi Yang, Zhengyou Wang, Shanna Zhuang, Hui Wang

**Affiliations:** ^1^School of Information Science and Technology, Shijiazhuang Tiedao University, Shaoxing, China; ^2^Hebei Key Laboratory for Electromagnetic Environmental Effects and Information Processing, Shijiazhuang 050043, China

## Abstract

Occlusion pedestrian detection is an important and difficult task in pedestrian detection. At present, the main method to deal with occlusion pedestrian detection usually adopts pedestrian parts or human body relationship methods. However, in the scene of crowd occlusion or severe pedestrian occlusion, only small parts of the body can be used for detection. Pedestrian parts or human body relationship methods cannot effectively address these issues. In view of the above problems, this paper abandoned the occlusion processing method of pedestrian parts or human body relationship. Considering that it is difficult to establish the relationship between parts and key points. The scale of visible parts of the occlusion pedestrian is small, and the scale of no occlusion pedestrian and occlusion pedestrian in the same picture is different. A multiscale feature attention fusion network named parallel feature fusion with CBAM (PFF-CB) is proposed for occlusion pedestrian detection. Feature information of different scales can be integrated effectively in the PFF-CB module. PFF-CB module uses a convolutional block attention module (CBAM) to enhance the important feature information in space and channel. A parallel feature fusion module based on FPN is used to enhance key features. The performance of the proposed module was tested on two common data sets of occlusion pedestrians with different occlusion types. The results show that the PFF-CB module makes a good performance in occlusion pedestrian detection tasks.

## 1. Introduction

With the rapid development of computer vision technology and the widespread application of artificial intelligence in various industries, pedestrian detection has become an important research topic. Obstructed pedestrian detection in pedestrian detection is an important and difficult task. The target of pedestrian detection is to obtain all the pedestrian location and size information in an image or video and label it with a rectangular box. If pedestrians are occluded, it will seriously affect the detection of pedestrians and the labeling of rectangular boxes, resulting in the inaccuracy of pedestrian detection. Pedestrian detection is the basis of various pedestrian-related research. Pedestrian detection technology has high application value and can be combined with pedestrian tracking, pedestrian reidentification [1] and other techniques for criminal investigation skid systems, unmanned systems [2], intelligent robot [3], and other fields. Therefore, accurate pedestrian detection is the basis of other pedestrian-based detection technologies, and effective processing of occluded pedestrian detection can effectively improve the accuracy of pedestrian detection.

At present, deep learning pedestrian detection algorithms are mainly based on convolutional neural networks (CNN). Pedestrian detection based on CNN is mainly divided into one- and two-stage networks. One-stage networks usually include YOLO [4], SSD [5], and DSSD. Two-stage networks usually include R-CNN, fast R-CNN [6], faster R-CNN, mask R-CNN [7], and cascade R-CNN [8]. This paper uses faster R-CNN and cascade R-CNN as the backbone in occlusion pedestrian detection.

In 2017, feature pyramid networks (FPN) [9] were applied to pedestrian detection feature extraction. After that, PANET [10] first proposed a bottom-up secondary fusion method. Both NAS-FPN [11] and bidirectional feature pyramid network (BiFPN) [12] seek an effective feature fusion mode in FPN. PFF-FPN [13] was proposed to handle different characteristics, and in this paper, PFF-FPN was used as a basic module. In this paper, we call it PFF.

For the occlusion pedestrian detection task, we divide it into target occlusion pedestrian detection and nontarget occlusion pedestrian detection.

At present, there are lots of methods of nontarget occlusion pedestrian detection. Tian [14] proposed the R-CNN depth-component method based on the partial detector structure, which consists of different partial detectors to form a depth-part detector. Zhou and Yuan [15] used the multilabel learning method on component detectors to improve the performance of detectors and reduce the calculation cost of some detectors by utilizing the correlation between component detectors. Wang et al. [16] optimized the loss function on the basis of faster R-CNN and proposed repulsion loss to effectively improve the detection accuracy of occlusion pedestrians. Zhang et al. [17] proposed OR-CNN (occlusion-aware R-CNN) on the basis of faster R-CNN, designed a new aggregation loss function, and proposed a new partial occlusion perception pooling layer to replace the original pooling layer to improve occlusion object detection. Zhou and Yuan [18] in 2017 proposed a multilabel learning method based on the common framework of partial detection and partial occlusion, which acquired partial occlusion mode through joint learning of partial detectors. Subsequently, in 2020 [19], a discriminant feature transformation was proposed, and a module was added to the faster R-CNN framework to enhance the feature separability of pedestrian and nonpedestrian samples for pedestrian detection and occlusion processing. Zhang et al. [20] proposed a simple and compact detection method for pedestrian occlusion based on the faster R-CNN structure. Through the visualization of the characteristics of each channel in the network, it is found that different channels have different responses to different body parts. Therefore, improving the suppression of useful channels can effectively improve the detection ability to block pedestrians. Zhou et al. [21] proposed that the pedestrian self-attention mechanism can effectively identify the pedestrian area and suppress background by splicer of semantic segmentation feature map and corresponding convolution feature map. Wu [22] proposed a tubular feature aggregation network to enhance the antijamming ability of the pedestrian detector by using the local time background of pedestrians in videos, so as to effectively identify obstructed pedestrians.

The solution of target occlusion pedestrian detection is mainly achieved by modifying nonmaximum suppression (NMS) algorithm. The classic NMS was initially applied to target detection by the r-CNN algorithm, strictly following the search for local maximum and suppression of nonmaximum elements. When there are two objects with a high degree of overlap in the image, classical NMS will filter according to the artificial threshold, resulting in missed detection. To solve such problems, Jan Hosang proposed a soft-NMS algorithm [23]. An attenuation function based on intersection over union (IOU) was used to reduce the confidence of the boundary box. The larger the IOU is, the greater the attenuation degree is so that the more sheltered pedestrians will not be missed. The NMS [24] further improves the NMS method and adds a positioning confidence prediction to make the position of the border with high classification confidence more accurate, thus effectively improving the detection performance. Adaptive-NMS [25] further optimized soft-NMS for the special application scenario of pedestrian detection in the crowd so that the NMS threshold in densely populated areas is larger, while the NMS threshold in sparse areas is smaller. Through a CNN network for density training, the optimal threshold is finally obtained to reduce the target occlusion error detection and leakage detection rate. Zheng proposed dou-NMS algorithm in Distal-Iou [26]. They believed that if the center point of adjacent frames is closer to the center point of the current maximum scoring frame, it is more likely to be a redundant frame. Therefore, candidate frames are screened through the center distance judgment. In addition to modifying the NMS algorithm, there are other methods to deal with dense crowd detection as follows. Zhang et al. [27] proposed an attribute-aware pedestrian detector to model semantic attributes of people in the way of advanced feature detection. In addition to typical semantic features such as center location, target scale, and offset, pedestrian-oriented attribute features are also introduced to encode high-level semantic differences between groups. Zhang et al. [17] proposed a new aggregation loss to force the approach of the suggestion box, and at the same time, a new partial occlusion perception area of interest unit was used to replace the occlusion perception R-CNN to improve the detection accuracy in the crowd. Zhou and Yuan [28] located the whole body and the visible part of a person by regression of two boundary boxes. Chu [29] proposed a method to make each proposal predict a group of related instances and set up NMS new technology to detect highly overlapping instances in crowded scenarios. Zhang [30] proposed dual-anchor RPN using the head to deal with the problem of crowd occlusion in human detection, simultaneously pairing the body and head parts, and achieving good results in the CrowdHuman data set.

 The main contributions of this work are as follows:

1. Parallel feature fusion with CBAM (PFF-CB) network was proposed for occlusion pedestrian detection. PFF-CB strengthens spatial useful features and useful channels through CBAM and parallel feature fusion. The fusion of different scale features is also processed.
2. For multiscale feature fusion, a parallel feature fusion method, PFF, is proposed. PFF strengthens important features through the corresponding feature fusion of three modules.
3. The PFF-CB module is verified in CrowdHuman and CityPersons data sets, and the results show that PFF-CB is effective in pedestrian detection. In CityPersons data set, the H index gets 47.01%. In CrowdHuman data sets, the MR^−2^ index gets 40.56%.

## 2. Related Work

### 2.1. Faster R-CNN and Cascade R-CNN

**Figure 1:**
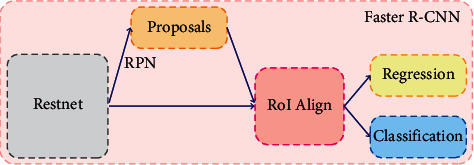
Faster R-CNN.

Faster R-CNN is the most widely used target detection structure network. The structure of faster R-CNN is shown in Figure 1.

**Figure 2:**
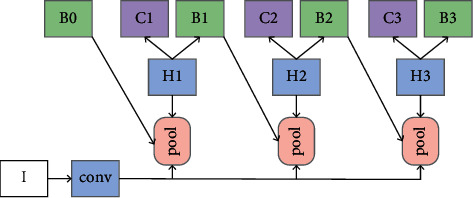
Cascade R-CNN.

In addition, in order to verify the effectiveness of the proposed module, this paper also carries out a simple verification on cascade R-CNN. The following is a brief introduction to cascade R-CNN, which is also a framework widely used at present. The purpose of continuously optimizing prediction results is achieved by cascading several detection networks. Different from ordinary cascading, several detection networks of cascade R-CNN [8] are trained on positive and negative samples determined by different IOU thresholds. The detailed structure is shown in Figure 2:

### 2.2. ResNet

**Figure 3:**
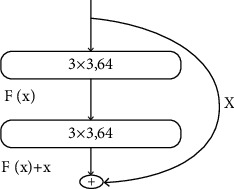
Residual structure.

The ResNet is a reference to the VGG19 network, which is modified on the basis, and the residual unit is added through the short-circuit mechanism. As the network deepens, the loss value will not be easily transmitted back, resulting in the disappearance of the gradient so that the network does not converge and does not achieve the best performance. The residual structure is to solve the problem of gradient disappearance; through the residual structure, the deep network can better trained. The structure residual is shown in Figure 3.

**Figure 4:**
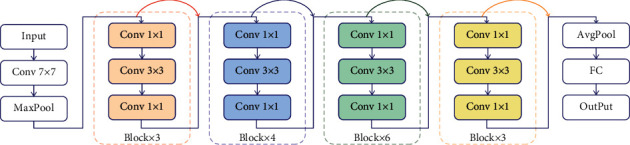
ResNet structure.

The structure of ResNet50 is shown in Figure 4.

### 2.3. FPN

**Figure 5:**
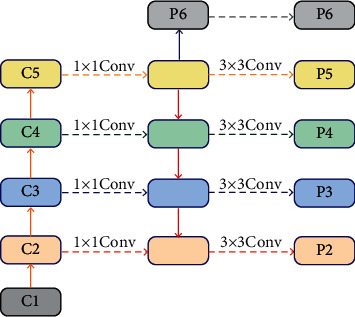
FPN structure.

FPN is consist of encoding and decoding blocks the cross-layer connection. FPN features are shown in Figure 5.

Different target sizes require different feature maps. C1 and C2 are shallow scale feature maps that are relatively friendly to small targets, while C4 and C5 are high-level semantic feature maps that are relatively friendly to large targets. However, the feature information of the key layer cannot be effectively emphasized, so the feature information of the key layer will be diluted by the feature information of the common layer, leading to information loss. Moreover, the current improved algorithms based on FPN such as PANET only reverse and repeat the fusion of information, and the current improved algorithms such as NAS-FPN [11] and BiFPN [12] only focus on the superposition of effective blocks. There is no selection and fusion between the key feature layers, so some important information may be lost.

### 2.4. CBAM

**Figure 6:**
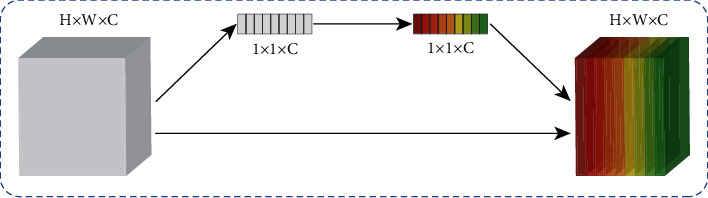
SE module.

The characteristic of human vision is that one does not obtain all information about an object at once. Instead, a series of local information about an object are gotten; then salient local information is selected to get the perception of object. CNN extracts a series of local features to get a whole scene. Recently, self-attention block has been extensively studied to improve the performance of CNNs in computer vision tasks. The self-attention block can recognize where the local information and which channel is more important to the computer vision task. To improve network performance, the self-attention block allows CNNs to focus on important information and suppress irrelevant information. Hu [31] proposed squeeze-and-excitation (SE) module shown in Figure 6.

**Figure 7:**
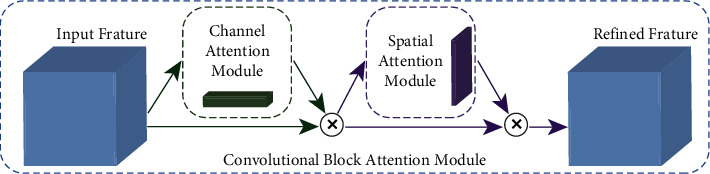
CBAM module.

The SE module, which only extracts channel information, guides CNNs to enhance meaningful feature channels and suppress useless feature channels. The SE module achieved high performance for image classification. Roy [32] introduced spatial squeeze and channel excitation (cSE), channel squeeze and spatial excitation (sSE), and concurrent spatial and channel squeeze and excitation (scSE) modules. The cSE module is the same as the SE module. For the segmentation task, the spatial information is more useful. Therefore, Roy proposed sSE module accounts for the spatial aspect. The spatial reweighting map is obtained by 1 × 1 convolution to enhance useful information and suppress irrelevant information in the spatial dimension. The scSE module consists of cSE and sSE modules. The feature maps are recalibrated by the scSE module in spatial dimension and channel dimension. Woo [33] proposed convolutional block attention module (CBAM). The CBAM block can easily be added into CNNs aggregates channel and spatial information to refine the input feature, which is a benefit to pedestrian detection. CBAM is shown in Figure 7.

## 3. Proposed Method

Considering that it is difficult to establish the relationship between parts and key points of the covered pedestrian, the scale of the visible part of the covered pedestrian is small, and the scale of the uncovered pedestrian and the covered pedestrian in the same picture is different, from the perspective of target scale. A multiscale feature attention fusion network (PFF-CB) is proposed for occlusion pedestrian detection. The PFF-CB network can effectively integrate feature information of different scales and use the CBAM to enhance the important feature information and the weight of key channels in space and channel, respectively. Parallel feature fusion module based on FPN was used to enhance key features.

### 3.1. PFF

In this paper, a parallel feature fusion (PFF) module is proposed to solve the fusion problem of key feature layers.

**Figure 8:**
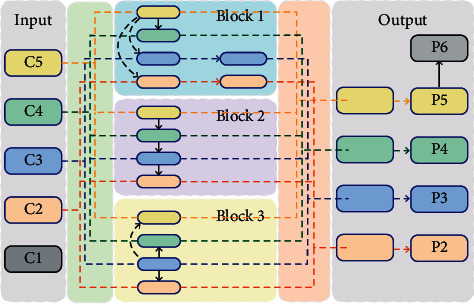
PFF structure.

The parallel feature fusion PFF structure proposed in this paper is shown in Figure 8.

**Figure 9:**
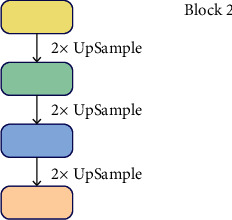
Block1 structure.

This module mainly includes three key modules: block1, block2, and block3. Block2 is the original FPN network. The structure of block2 is shown in Figure 9.

In order to fully consider the feature scale difference. We extended block3 to get block4.

**Figure 10:**
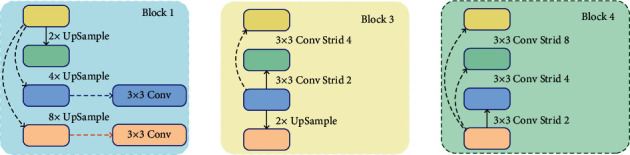
Block1, block3, and block4 structure.

The structure of block1, block3, and block4 proposed in this paper is shown in Figure 10.

Block1 selects C5 as the basic feature. After 1 × 1 convolution, 2 times upsample, 4 times upsample, and 8 times upsample are performed to obtain the same feature scale as C4, C3, and C2 and correspond to C4, C3, and C2. After feature 1 × 1 convolution, features are added pixel by pixel for feature fusion. The features obtained by pixel-by-pixel addition after 1 × 1 convolution with C3 and C2 features are subjected to a 3 × 3 convolution operation to obtain features enhanced by block1.

Block3 selects C3 as the basic feature. After 1 × 1 convolution, 2 times upsample and C2 feature 1 × 1 convolution are performed, and the feature is added pixel by pixel for feature fusion, and a 3 × 3 volume with a stride of 2 is performed. A 3 × 3 convolution with a product and a stride of 4, and the corresponding C4 and C5 features 1 × 1 convolution features are added pixel by pixel for feature fusion. In this way, important features are obtained through block3 enhancement.

Block4 selects C2 as the basic feature. After 1 × 1 convolution, 3 × 3 convolution with stride 2, 3 × 3 convolution with stride 4, and 3 × 3 convolution with stride 8 are performed. The product is combined with the corresponding C3, C4, and C5 features after 1 × 1 convolution to perform pixel-by-pixel addition for feature fusion. Thereby, the features after block4 enhancement are obtained.

After obtaining extracted by the three features, the corresponding features are added pixel by pixel to get the feature, and then the features P2, P3, P4, and P5 are obtained through 3 × 3 convolution. In order to better detect large target pedestrians, this paper increases the receptor field again and carries out the maximum pooling operation on P5 to get the P6 feature. At this point, the final features of the PFF module have been obtained, namely, P2, P3, P4, P5, and P6.

Since the C3 and C2 features are relatively friendly to small targets, the enhanced features of block3 and block4 will be beneficial to the detection of small targets, but the C2 feature used in block4 contains many noise points. Therefore, the experiments in this paper first compare and verify block3 and block4 to prove the impact of block3 and block4 on network performance.

### 3.2. PFF-CB

**Figure 11:**
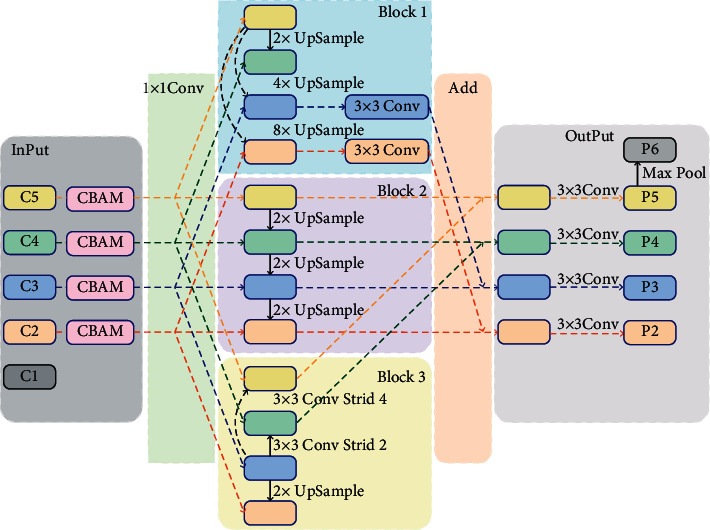
CBAM refined PFF features.

In order to better extract the feature map, we refined the input features after the PFF input features. We added a lightweight attention module (convolutional block attention module, CBAM) to the input features. Channel and spatial attention can effectively refine feature maps for pedestrian targets so that PFF can better process the extracted feature maps. The detailed structure diagram of the added location of the CBAM module is shown in Figure 11.

We added the CBAM module to the C1–C5 features after feature extraction and optimized the extracted features again so that the occluded and occluded pedestrian features in the occluded pedestrian can be better included in the C1–C5 feature map.

From Figure 7, it can be seen that CBAM attention includes two independent attention submodules: channel attention module (CAM) and spatial attention module (SAM). Enter the acquired feature maps into two modules, CAM and SAM, for channel attention and spatial attention. It can effectively process the feature map, which is the refinement mentioned in this article.

The attention of occluded and blocked pedestrians through the CBAM channel can better focus on the relevant components of the pedestrian so that the important channel feature map can be enhanced.

The spatial attention of CBAM can effectively enhance the pedestrian position information, so CBAM added to the attention module has a certain effect on the detection of occluded pedestrians. After theoretical analysis, the feature maps C1–C5 after CBAM attention has more useful features, so we add the extracted features to the CBAM attention for feature processing after ResNet extracts the features, so as to obtain a more suitable channel for pedestrian detection and the characteristic map of the space. In order to verify the usefulness of CBAM in follow-up experiments, we conducted separate experiments to verify it.

### 3.3. Application of PFF Module in Network Framework

**Figure 12:**
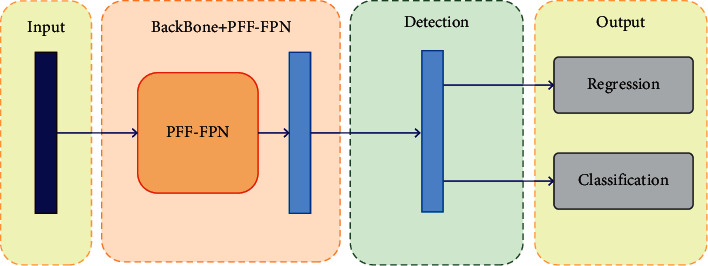
Pedestrian detection network added PFF.

PFF module can be used in lots of networks. The structure of PFF used in networks is shown in Figure 12.

PFF replaces the original FPN without affecting the subsequent detection network. Therefore, PFF is a very convenient replacement module. In subsequent experiments, we will also apply PFF in different network frameworks for experimental comparison.

## 4. Experiments

This section mainly introduces the related content of the experiment, including data set, experimental environment, verification index, comparison data, and so on.

### 4.1. Data Sets

**Figure 13:**
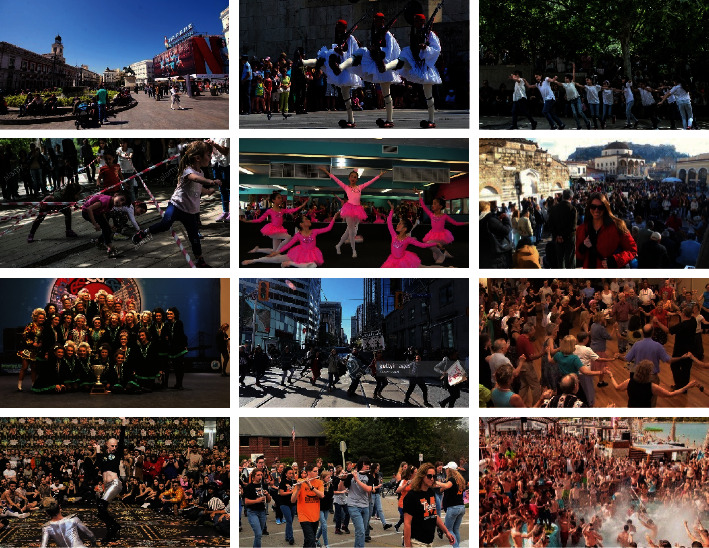
CrowdHuman data sets.

In this paper, CrowdHuman [29] and Citypersons data sets are used for experimental verification of the occluded pedestrian detection data set. The CrowdHuman data set has a large amount of data. There are about 23 people in each image in the CrowdHuman data set. Therefore, there is mutual occlusion between pedestrians, which can also be called mutual occlusion between targets. Each human body instance has a head, a visible area of the human body, and a human body bounding box. Selecting the CrowdHuman data set can effectively verify the occlusion pedestrian detection between targets. The occlusion picture of the CrowdHuman data set is shown in Figure 13.

The CityPersons data set is a subset of Cityscape and contains only personal annotations. There are 2,975 pictures for training and 500 and 1,575 pictures for verification and testing, respectively. The average number of pedestrians in the image is 7, providing visual area and full-body annotations.

CityPersons contains more real-world objects to block pedestrians, such as bicycles to block pedestrians, walls to block pedestrians, and cars to block pedestrians. The occlusion of pedestrians by many objects can also be called nontarget occlusion of pedestrians.

**Figure 14:**
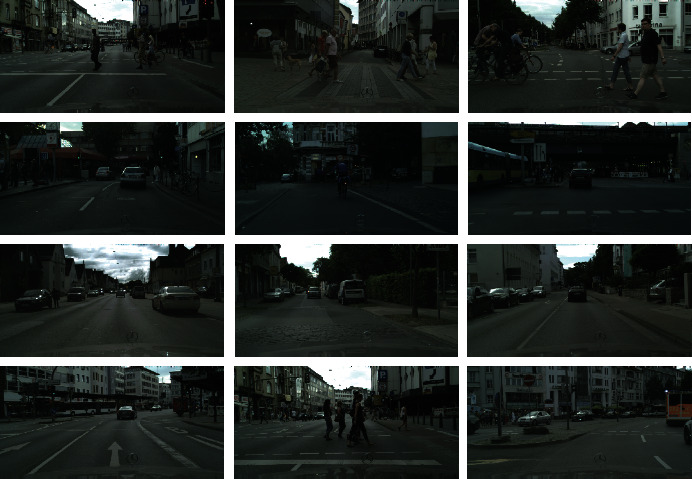
CityPersons data sets.

Therefore, the selection of CityPersons can effectively verify the performance of objects for occlusion detection. CityPersons partial occlusion picture is shown in Figure 14.

The CrowdHuman and CityPersons data sets are common pedestrian detection data sets with a relatively rich amount of data and can fully train the model. Secondly, the CrowdHuman and CityPersons data sets occlude more pedestrians and contain different occlusion situations. For example, CrowdHuman mainly includes mutual occlusion between pedestrians, and CityPersons mainly includes the occlusion of pedestrians by objects, and the two data contain rich small targets at the same time. And the large target information can effectively verify the effectiveness of the proposed method in the detection of occluded pedestrians.

### 4.2. Environment Configuration

The software environment and hardware environment in this paper are shown in Tables 1 and 2.

### 4.3. Evaluation Metrics

Three evaluation metrics are used to verify experimental results.

Averaged precision (AP)

(1) $$\begin{array}{c} \text{AP}=\int_{0}^{1}p\left(r\right)\text{d}r, \end{array}$$

where *p* is accuracy *r* is the recall rate

(2) $$\begin{array}{c} p=\frac{tp}{\left(tp+fp\right)},r=\frac{tp}{\left(tp+fn\right)}. \end{array}$$

Another evaluation metrics

(3) $$\begin{array}{c} {\text{MR}}^{-2}=\frac{fp}{n}. \end{array}$$

In addition, in the data set of CityPersons, the visibility of pedestrians was divided into Bare [0.95, 1], Reasonable [0.65, 1], Partial [0.65, 0.9], Heavy [0, 0.65] (H), and Reasonable Heavy [0.2–0.65] (R-H) subsets from high to low. These indicators are represented using MR^−2^ so the lower the value, the better the performance. The most common ones are Reasonable and Heavy, which can be used to monitor the indicators under light occlusion and Heavy occlusion; the lower the parameters, the better the performance.

### 4.4. Analysis of Experimental Results

There are the following groups of experiments to verify the three feature processing blocks, PFF module, and PFF-CB network proposed in this chapter. First, verify the performance comparison of PFF-3 and PFF-4 to determine the specific feature processing block to be adopted later. Secondly, verify the improvement effect of the PFF-FPN module on the basic network. Next, verify the effect of embedding the CBAM attention mechanism in the PFF-FPN module on the basic network. Finally, the performance improvement of the PFF-CB network in the task of occluding pedestrian detection is verified. In order to fully verify the performance improvement of the PFF-CB network under different occlusion conditions, each group of experiments uses the CrowdHuman and CityPersons data sets for verification.

The first experiment verifies the block3 and block4 modules and names them as PFF-3 (original FPN, block1, and block3 are processed in parallel) and PFF-4 (original FPN, block1, and block4 are processed in parallel). The result is shown in Table 3:

Compared with PFF-4, the AP index of PFF-3 increased by 0.19%, and MR^−2^ (the lower the better) decreased by 0.67%. The performance of PFF-3 is slightly better than that of PFF-4, so we use the PFF-3 structure in subsequent experiments.

To verify the experimental results of the PFF module, we adopted faster R-CNN and cascade R-CNN networks. The results of the experiment are shown in Table 4:

In faster R-CNN at CrowdHuman data set, the AP index of PFF gets 86.61%, and the MR^−2^ index reaches 42.39%, which is better than the AP index and the MR^−2^ index of FPN.

In cascade R-CNN at CrowdHuman data set, the AP index of PFF gets 87.13%, and the MR^−2^ index reaches 40.56%, which is better than the AP index and the MR^−2^ index of FPN.

We also compared PFF with the most advanced methods in the CrowdHuman data set. The network with the PFF module also has better performance and achieves the best results in the MR^−2^ index. The experimental results are shown in Table 5:

Faster R-CNN, adaptive NMS, PS R-CNN, iterdet, iterdet2, and faster R-CNN + PFF are all improved and tested based on faster R-CNN. It can be seen that the MR^−2^ index of the original faster R-CNN under the CrowdHuman data set is 50.49%, and in the adaptive NMS method, the MR^−2^ index reaches 49.73%. In iterdet and iterdet2 methods, MR^−2^ reached 49.12% and 49.22%, respectively. By adding the method proposed in this paper to faster R-CNN, the MR^−2^ index reached 42.39%, compared with the original method faster R-CNN network MR^−2^ (the lower the better) decreased by 8.1%.

Compared with other networks, it decreased by 7.34%, 6.73%, and 6.83%, respectively. Experiments show that PFF can improve the missed detection rate of blocked pedestrians under the framework of faster R-CNN. Other methods include noh-nms, crowddet, beat R-CNN, defcn(poto + 3DMF + aux, ResNet), cascade R-CNN + FPN, and cascade R-CNN + PFF.

These methods have MR-2 indexes of 43.9%, 41.1%, 48.9%, 43.0%, and 40.56%, respectively, in CrowdHuman data set. It can be seen that cascade R-CNN network achieves the optimal effect after adding our PFF module, which is 40.56%.

We also conducted experimental verification on the CityPersons data set. The experimental results are shown in Table 6.

**Figure 15:**
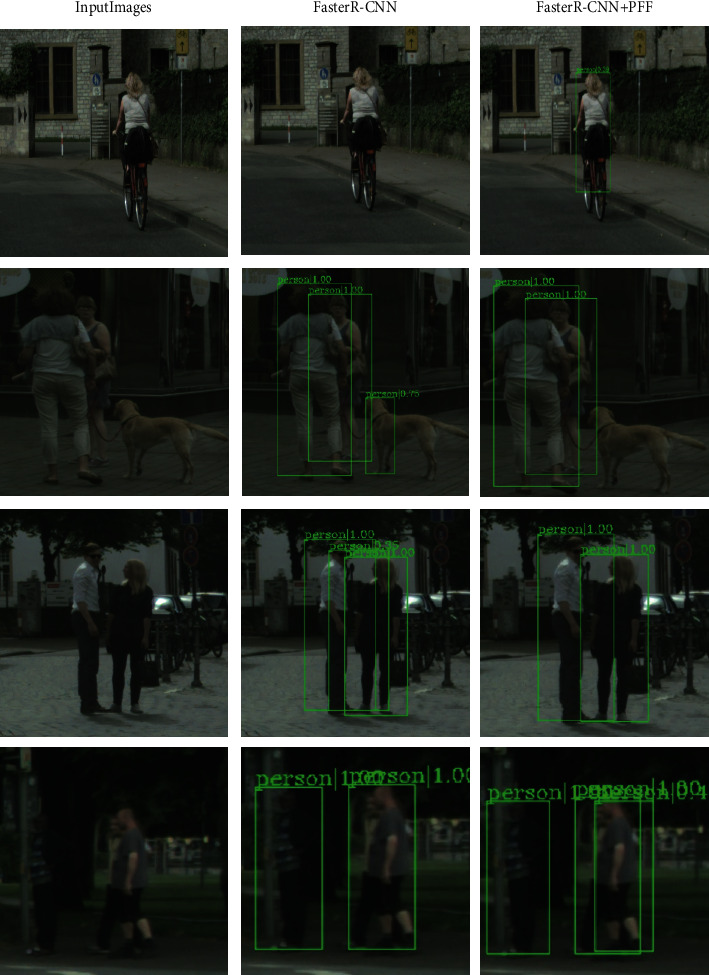
Detection results in faster R-CNN in CityPersons.

In CityPersons data set, under the faster R-CNN baseline network, PFF in R-H (the lower the better) index reached 39.80%, which was 0.75% lower than FPN. In the cascade R-CNN baseline network, PFF at R-H (the lower the better) reached 38.64%, 2.12% lower than FPN. Through the above experiments, it is proved that the PFF module has a better effect on the baseline network in the case of mutual occlusion between pedestrians and occlusion between objects. As the PFF module is greatly improved in the CityPersons data set, a simple visual verification is carried out on the CityPersons data set, and errors are classified. First, it was compared with the faster R-CNN baseline network. The result can be seen clearly in Figure 15.

Figure 15 includes part of the original pictures of the test set, pictures detected by faster R-CNN, and pictures detected by faster R-CNN after adding PFF. As can be seen from the above table, the first picture has bicycles occluding pedestrians. The original baseline network faster R-CNN cannot effectively detect pedestrians occluded by bicycles. After adding the PFF module, we successfully detected the pedestrians. Pedestrians are blocked by bicycles.

The original faster R-CNN in Figure 2 has a false detection and detects the dog as a pedestrian. After adding the PFF module, the useful features are effectively integrated, and the dog features are eliminated, thereby effectively detecting the pedestrian target. In the third picture, due to the close distance between two people, the original.

Faster R-CNN produced a false detection frame between the two people, and the network after adding PFF successfully avoided this problem. In the fourth picture, there is mutual occlusion between pedestrians. The original faster-R-CNN detects the mutual occlusion of pedestrians as a pedestrian, which leads to the occurrence of missed detection. The network after adding the PFF module can effectively separate the two pedestrians with each other, and the pedestrian targets in dense obscuration can be successfully detected.

**Figure 16:**
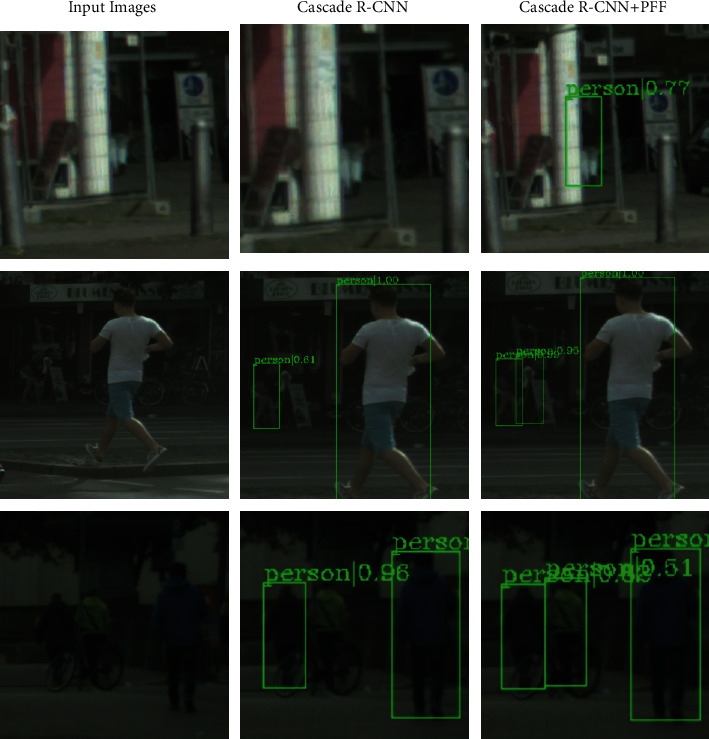
Detection results in cascade R-CNN in CityPersons.

Figure 16 includes some original pictures of the test set, pictures detected by cascade R-CNN, and pictures detected by cascade R-CNN after adding PFF. As can be seen from the above table, the first picture has the occlusion of pedestrians by the wall. The original baseline network cascade R-CNN cannot effectively detect the pedestrians occluded by the wall, but we successfully detected the pedestrian after adding the PFF module pedestrians obscured by the wall. In Figure 2, the original cascade R-CNN could not detect half-length pedestrians blocked by billboards. After adding the PFF module, it successfully detected half-length pedestrians blocked by billboards. The baseline network cascade R-CNN cannot effectively detect pedestrians obscured by bicycles, but after, we added the PFF module, and we successfully detected pedestrians obscured by bicycles.

It can be proved that our proposed module has improved the data set index of occlusion of pedestrians by the CityPersons object. The CrowdHuman data set is mainly aimed at the mutual occlusion between pedestrians and the CityPersons data set is more inclined to the occlusion of people by objects. It can be seen that the module proposed in this paper is effective in detecting the occlusion of pedestrians.

In order to further study the performance of this module, we also carried out other experiments to verify it.

The PFF module has high requirements for the feature maps obtained from ResNet, and the high-quality feature maps can give full play to the effect of PFFN. Therefore, we improved the feature extraction and added the CBAM attention module to PFFN, so as to obtain high-quality feature maps.

In order to verify the final effect of the proposed module, we carried out the final experiment on the basis of faster R-CNN and CBAM added the proposed PFF. The experimental results are shown in Table 7.

The experimental results show that the proposed module is 0.95% lower than the faster R-CNN and ResNet50 and CBAM in the H index (the lower the better) and 1.7% lower than the faster R-CNN and ResNet50 of the basic network, which fully proves the effectiveness of the proposed module.

We also compare PFF with the most advanced methods in the CityPersons data set. The network with the PFF module also has better performance and achieves the optimal result in H (the lower the better) index. The experimental results are shown in Table 8.

## 5. Conclusion

An effective PFF-CB module is proposed in this paper, which is based on PFF and CBAM, PFF-CB module is aiming at the problem of characteristics of pedestrian detection targets extraction. PFF method is proposed to enhance the important features. In order to make full use of PFF, the CBAM attention module is added to the feature extraction network. The experiments were carried out on the CrowdHuman data set and CityPersons data set, In cascade R-CNN, the PFF module gets the best performance in the CrowdHuman data set, which the MR^−2^ index gets 40.56%. In CityPersons data set, the results show that the PFF-CB module proposed in this paper can improve the detection efficiency. PFF-CB added to faster R-CNN in CityPersons data set, and the H index got 47.01% and achieved the optimal effect.

## Figures and Tables

**Table 1:** Hardware configuration.

Hardware	Configuration
CPU	Intel (R) I7 CPU 8700K
GPU	2080Ti
HDD	480 GB

**Table 2:** Software environment.

Software	Configuration
System	Ubuntu 18.04
Libraries	Cudnn, CUDA, OpenCV, Cython, Numpy
Language	Python
Framework	Pytorch 1.4.0, Megengine 0.3.1, mmdetection

**Table 3:** The result of PFF-3 and PFF-4

Module	AP (%)	MR^−2^ (%)
Faster R-CNN + FPN	85.80	42.90
Faster R-CNN + PFF-3	**86.61**	**42.39**
Faster R-CNN + PFF-4	86.42	43.06

**Table 4:** The result of PFF in faster R-CNN and cascade R-CNN in CrowdHuman data sets.

Module	AP (%)	MR^−2^ (%)
Faster R-CNN + FPN [29]	85.80	42.90
Faster R-CNN + PFF	**86.61**	**42.39**
Cascade R-CNN [29]	85.60	43.00
Cascade R-CNN + PFF	**87.13**	**40.56**

**Table 5:** Compared PFF with the most advanced methods in the CrowdHuman data set.

Module	MR^−2^ (%)
Faster R-CNN	50.49
Adaptive NMS [25]	49.73
IterDet [34]	49.12
IterDet2 [34]	49.22
Faster R-CNN + PFF (ours)	**42.39**
NOH-NMS [35]	43.90
CrowdDet [36]	41.40
DeFCN (POTO + 3DMF + AUX)	48.90
Cascade R-CNN + FPN	43.00
Cascade R-CNN + PFF (ours)	**40.56**

**Table 6:** Experimental results about faster R-CNN and cascade R-CNN in CityPersons.

Module	R-H (%)
Faster R-CNN + FPN	40.55
Faster R-CNN + PFF	**39.80**
Cascade R-CNN	40.76
Cascade R-CNN + PFF	**38.64**

**Table 7:** Final experimental results in CityPersons.

Module	R-H (%)
Faster R-CNN + FPN	40.55
Faster R-CNN + PFF	39.80
Faster R-CNN + PFF-CB	**38.85**

**Table 8:** Experimental results in CityPersons data set

Module	H (%)
Rep loss [16]	56.90
OR-CNN [17]	55.70
TLL	53.60
TLL + MRF	52.00
ALFNet [37]	51.90
CSP	49.30
NOH-NMS [35]	53.00
Faster R-CNN	49.24
Beat R-CNN [38]	47.10
PRF-Ped [39]	47.30
Faster R-CNN + PFF (ours)	47.29
Faster R-CNN + PFF-CB (ours)	**47.01**

## Data Availability

Data are available on request from the corresponding author.
